# Heritable human genome editing: correction, selection and treatment

**DOI:** 10.1093/medlaw/fwae003

**Published:** 2024-03-21

**Authors:** Rosamund Scott

**Affiliations:** Centre of Medical Law and Ethics, The Dickson Poon School of Law, King’s College London, UK

**Keywords:** Heritable human genome editing (HHGE), Prospective parents, Future child, Correction, Selection, Treatment

## Abstract

Heritable human genome editing (HHGE) to correct a nuclear gene sequence that would result in a serious genetic condition in a future child is presented as ‘treatment’ in various ethics and policy materials, and as morally preferable to the ‘selection’ practice of preimplantation genetic testing (PGT), which is subject to the disability critique. However, whether HHGE is ‘treatment’ for a future child, or another form of ‘selection’, or whether HHGE instead ‘treats’ prospective parents, are now central questions in the debate regarding its possible legalisation. This article argues that the idea of ‘treatment’ for a future child is largely a proxy for ‘seriousness of purpose’, intended to distinguish HHGE to avoid serious genetic conditions from less obviously justifiable uses; that HHGE is best understood, and morally justified, as a form of ‘treatment’ for prospective parents who strongly desire an unaffected genetically related child and who have no, or poor, options to achieve this; that HHGE would be morally permissible if consistent with that child’s welfare; that legalisation is supportable with reference to the right to respect for private and family life under Article 8 of the European Convention on Human Rights; and that HHGE is morally distinguishable from PGT.

## I. INTRODUCTION

At the second international summit on heritable human genome editing (HHGE) in 2015, Dr He Jiankui announced that he had conducted HHGE on the embryos of subsequently born twins, supposedly to create resistance to a form of human immunodeficiency virus (HIV).^1^ This was widely condemned. The Concluding Statement of the summit’s Organising Committee criticised the highly premature nature of this intervention, noting that it ‘was irresponsible and failed to conform with international norms’.^2^ In due course there were calls in some quarters for a moratorium on the use of HHGE pending an international framework entailing certain commitments, including international consultation and a finding of a ‘broad societal consensus’ prior to any national decisions.^3^ Meanwhile, others honed various arguments in opposition to the legalisation of HHGE: first, that since embryos have to be created to be edited, HHGE does not ‘treat’ future children, is ultimately just another way to ‘select’ who comes into being, and is thus equally subject to the disability critique of selection practices such as preimplantation genetic testing (PGT), in which embryos are tested for a serious genetic condition and typically discarded if found to be affected; and second, that since prospective parents at risk of having a genetically related child with a serious genetic condition have alternative ways to have a child—such as by adopting or by using donor gametes together with in vitro fertilisation (IVF)—HHGE is also unnecessary.^4^ Thus, a significant strand of opposition to the legalisation of HHGE (if and when established as sufficiently safe for clinical use) has focused on critique of the concept of HHGE as ‘treatment’ in ethics and policy debates. Such critique supposedly undermines or negates the argument that the use of HHGE would be morally justifiable in answer to a therapeutic need, or that it would morally improve on established legal ways to avoid the birth of a child with a serious genetic condition.

In this article, I consider use of the concept of ‘treatment’ or ‘therapy’ in the relevant international instruments, UK law, UK and international policy materials, and the ethics literature. I address whether, and if so in what ways, these concepts should be used in the further consultation processes and policy debates that will necessarily precede any legalisation of HHGE. I argue that in various sections of the ethics and policy debates so far, the idea of ‘treatment’ for a future child is really a proxy for ‘seriousness of purpose’, intended to distinguish HHGE to avoid serious genetic conditions from less obviously justifiable uses, but that such use is inappropriate as regards a future child, and that ‘correction’ is the better term. This does not mean that ‘treatment’ is irrelevant to the debate. To the contrary, I argue that HHGE is best understood, and morally *justified*, not as treating a future child, but rather as treating the prospective parents who desire a genetically related child unaffected by a serious genetic condition but have no, or poor, options to achieve this without HHGE. I also argue that such HHGE (if and when established as sufficiently safe) would be morally *permissible* if consistent with that child’s welfare. Furthermore, analysis of the ways in which HHGE may be used in initial clinical practice shows that, with reference to the disability critique of selection practices, HHGE *is* morally distinguishable from PGT. This article is structured as follows.

In Section II, by way of background, I introduce the leading international instruments of relevance to HHGE and note the scope for interpretation in them as regards HHGE. I also outline the key provisions of relevant UK law.

In Section III, I consider the presentation of HHGE as ‘treatment’, mostly for a future child but sometimes for the prospective parents, in UK policy discussion and international policy reports. As regards the future child, I suggest that the preferable term is ‘correction’ rather than ‘treatment’; the former is prevalent in the report of the World Health Organization (WHO) Expert Advisory Committee on Developing Global Standards for Governance and Oversight of Human Genome Editing (the WHO report).^5^

Thereafter, Section IV turns to the ethics literature. I first note the presentation of HHGE as ‘treatment’ of a future child in early literature, and then consider the challenge that HHGE is not ‘treatment’, is ‘unnecessary’, and should therefore not be legalised. With reference to the ethics literature, I consider how we should understand ‘treatment’ in relation to HHGE. I also suggest possible explanations for the prevalence of the term ‘treatment’ in connection with the future child in policy materials: for instance, to distinguish HHGE from the selection practice of PGT (and thereby to distance it from the disability critique) or from concerns about ‘design’, and ‘enhancement’. As regards these latter concerns, I argue that ‘treatment’ is really a proxy for ‘seriousness of purpose’, notwithstanding that there may be serious ‘enhancement’ purposes.

In Section V, recognising that the interest in HHGE arises because of the desire of prospective parents at risk of having a child with a serious genetic condition for a genetically related child, I discuss the broadly supportive approach to this desire, and consideration of the alternatives for prospective parents, in the international policy reports.^6^ The section then considers how this approach can be developed in law, with particular reference to the right to respect for private and family life under Article 8 of the European Convention on Human Rights (ECHR). The analysis in this section shows that, morally and legally speaking, HHGE is best understood, and *morally justified*, as a form of treatment for *prospective parents—*preventing the transmission of a serious genetic condition to offspring, thereby enabling them, following a successful pregnancy, to have a genetically related child unaffected by a serious genetic condition.

Section VI then addresses the way HHGE may be used in two categories of case in initial clinical practice, as outlined in one of the international reports. Since HHGE would be accompanied by various testing and selection practices, this section analyses the purpose of these, and how they would compare with those at stake in PGT. It also considers how we should understand the focus, in many policy reports, on the welfare of the future child. I argue that ‘welfare’ should not be understood in relation to ‘treatment’, but instead as relevant to the *moral permissibility* of HHGE, with particular regard to the concept of risk.

My focus is on serious monogenic conditions because HHGE, if used in clinical practice, may never progress beyond these given both a lack of scientific knowledge and a more complex ethical case for the justification of broader use, which is beyond my current scope.^7^ As regards terminology, I use the term ‘serious genetic condition’ to encompass either disease or impairment, and ‘affected’ when referring to an embryo or person affected by such a condition.

## II. BACKGROUND—INTERNATIONAL AND DOMESTIC LAW

### A. International instruments

Several international instruments are relevant by way of background. None of them is clear as regards the legal standing of HHGE and so all are open to interpretation.

The first is the 1997 United Nations Educational, Scientific and Cultural Organization (UNESCO) Universal Declaration on the Human Genome and Human Rights (UNHGHR). Section A is entitled ‘Human Dignity and the Genome’: Article 1 highlights the idea of the human genome underlying humans’ ‘unity … diversity and dignity’, while Article 2 refers to everyone having a ‘right to respect for their dignity’ and rights ‘regardless of their genetic characteristics’. The prevalence of ‘dignity’ in the declaration has been noted by Deryck Beyleveld and Roger Brownsword.^8^ Turning to the concept of ‘treatment’, Article 5(a) states (in part) that ‘[r]esearch, *treatment* or diagnosis affecting an individual’s genome shall be undertaken only after rigorous and prior assessment of the potential risks and benefits’, and consideration of national laws.^9^ Whether HHGE is ‘treatment’ is of course a key question to which I turn in Section IV.

Turning to the section which concerns implementation, Article 24 refers to the International Bioethics Committee (IBC) of UNESCO making recommendations regarding ‘the identification of practices that could be contrary to human dignity, such as germ-line interventions’. This *may* imply that Article 5 should be interpreted as referring only to somatic interventions (which relate to a cell in the human body that is not a germ cell) but this is not clear. Whether or not germline interventions are ‘contrary to human dignity’ raises the question of what is meant by ‘dignity’. As Timothy Caulfield and Roger Brownsword observe, at some points in the declaration ‘dignity’ is associated with ‘empowerment’, aligned with autonomy, and at others with ‘restraint’,^10^ aligned with ‘general condemnation’ of new technologies, a use of ‘dignity’ that has problematic implications for policy-making in pluralistic societies.^11^ As regards Article 24 itself, the Deutscher Ethikrat, the German Ethics Council, observes that ‘no explicit violation of human dignity is identified nor is a prohibition of germline intervention explicitly stated’.^12^ In this light, the UK Government’s decision that the legalisation of mitochondrial replacement techniques (MRTs, which entail the substitution of mitcohondria either in eggs or embryos to avoid serious genetic conditions in future people)^13^ would *not* be contrary to human dignity under Article 24 is defensible. The same decision could be made in relation to HHGE, supported by the point that editing an embryo does not ‘reduce individuals to their genetic characteristics’, and thus would not be contrary to Article 2. However, not all will agree.

An approach that is likewise subject to interpretation is taken in the Council of Europe’s 1997 Convention on Human Rights and Biomedicine, the Oviedo Convention, described by the Council as a ‘framework Convention aiming at protecting the dignity and identity of all human beings’.^14^ The UK is not a signatory. Article 13 states that ‘an intervention seeking to modify the human genome may only be undertaken for *preventive*, diagnostic or *therapeutic* purposes and only if its *aim is not to introduce any modification in the genome of any descendants*’.^15^ As regards the terms ‘preventive’ or ‘therapeutic’, these were clarified in late 2022 by the Steering Committee for Human Rights in the Fields of Biomedicine and Health (CDBIO), which stated: ‘[A] preventive purpose … will aim at avoiding the occurrence of a disease or disorder’; and ‘a therapeutic purpose … will aim at controlling symptoms of a disease or disorder, slowing or reversing its progression, or providing a cure to a disease or disorder, for example by removing its underlying cause’.^16^ The clarification makes no reference to HHGE and so its status remains unclear.^17^ Whether editing an embryo can be understood as ‘therapeutic’, for instance if HHGE were considered ‘pre-emptively therapeutic’,^18^ will be discussed in Section IV. The idea of HHGE as ‘preventive’ appears plausible, though requires further discussion in due course.

Turning to the phrase ‘only if its aim is not to introduce any modification in the genome of any descendants’, it is the *embryo* that is the subject of editing, and the aim of HHGE is to edit its genome. In contrast, ‘modification’ of any descendants of the person born from that embryo could be considered an ‘effect’, rather than an ‘aim’. In any event, questions also arise as to the meaning, and significance, of ‘modification’.^19^ The Explanatory Report notes that there may be ‘great benefit for humanity’ from relevant developments and stresses, regarding the possibility of ‘misuse’, that ‘[t]he ultimate fear is of intentional modification of the human genome so as to produce individuals or entire groups endowed with *particular characteristics and required qualities*’.^20^ This may reflect a worry about eugenics, historically aligned with actions of the state, rather than the private choices of individuals to avoid, by some means, the birth of a child with a serious genetic condition.^21^ This concern was reiterated in a 2015 statement of the Council of Europe’s Committee on Bioethics, which undertook to consider recent developments in the light of the principles of the Convention.^22^ In short, it is arguable that HHGE to avoid a serious genetic condition in a future child would be compatible with the Convention.

While the UK is no longer part of the European Union, Article 3 of the EU Charter of Fundamental Rights, regarding the ‘Right to Integrity of the Person’, prohibits ‘eugenic practices, in particular those aiming at the *selection* of persons’.^23^ An explanatory note clarifies that this is intended to refer to ‘programmes’ including ‘campaigns for sterilisation’,^24^ so it is doubtful that private decisions to employ germline editing would have been intended to be caught by this.^25^

As has been seen, the existing instruments are open to interpretation. They are likely to be interpreted differently depending on the view of HHGE that may be taken. Whether there will ever be a formal international agreement on HHGE is not yet clear. The WHO report of 2021 notes the need for governance of HHGE both at ‘national … and transnational levels’, understood ‘as including the norms, values and rules of the processes through which public affairs are managed so as to ensure transparency, participation, inclusivity and responsiveness’.^26^ The report observes that the Committee ‘encourages but cannot mandate a coordinated global approach’ and that, in the absence of such an approach, ‘different jurisdictions, with different political regimes and cultural, historical, and religious contexts, will likely … [prefer] different regulatory approaches’.^27^ More generally, the leading policy reports discussed in due course are not particularly optimistic about the possibility of a formal international agreement, as others have noted with reference to the 2021 report of the WHO and the 2020 report of the National Academy of Medicine, National Academy of Sciences, and the Royal Society (the International Commission report).^28^ Nonetheless, the reports highlight the scope for various forms of international coordination, cooperation and communication, and the International Commission report recommends, for example, an International Scientific Advisory Panel.^29^

### B. Domestic law

As for domestic law, under the Human Fertilisation and Embryology (HFE) Act 1990 (as amended by the HFE Act 2008) (the HFE Act (as amended) or the amended Act), HHGE is currently prohibited by means (in part) of the device of what is—and is not—a ‘permitted’ embryo or gamete: by section 3(2) only these can be placed in a woman. What is *not* permitted is an egg, sperm, or embryo whose nuclear DNA has been ‘altered’,^30^ together with an embryo to which a ‘cell has been added… other than by division of the embryo’s own cells’.^31^ An exception in section 3ZA(5), coupled with subsequent regulations, allows for the use of MRTs.^32^ These are licensable as ‘treatment services’, defined as ‘medical, surgical or obstetric services provided to the public or a section of the public for the purpose of assisting women to carry children’.^33^ As for who the recipient of such treatment is, this is not fully clear, just as it was not in the policy debate regarding MRTs.^34^ Whether this should be understood as the woman who is involved in the relevant medical procedures will be considered in various ways in Sections III to VI. Of note, also PGT is licensable as an activity ‘in the course of providing *treatment* services’, which may include ‘testing … embryos’.^35^ Indeed, the only licences that can currently be granted under the Act are for ‘authorising activities in the course of providing treatment services’, or ‘non-medical fertility services’, or for storage or research.^36^ So, any clinical procedure must be classed as ‘treatment’ (or a ‘fertility service’) or else no licence for it can be granted. The same will go for HHGE, in the absence of some change to section 11 on the class of possible licences. Thus, ‘treatment’, coupled in the Act with ‘services’ and encompassing ‘activities’ that are part thereof, is of considerable legal significance, notwithstanding the lack of clarity about the recipient—apart, that is, from the stipulation that such services are for the ‘public’ or part thereof, to ‘assist … women to carry children’.

Having noted the use and place of ‘treatment’, ‘therapeutic’ and ‘preventive’ in relevant international instruments and domestic law, I now turn to consider the various understandings of ‘treatment’, as regards HHGE, in the policy debate, including in the international reports.

## III. THE POLICY DEBATE—UNDERSTANDINGS OF ‘TREATMENT’

‘Any words that are chosen to describe a new technology have an impact on the discourse about it’: so said the European Group on Ethics in Science and New Technologies (EGESNT) with respect to genome editing in 2021.^37^ Indeed, although in 2016 a UK Parliamentary Office of Science and Technology (POST) Postnote referred to HHGE as ‘therapy’,^38^ a 2019 report of the House of Commons Science and Technology Committee (HCSTC) identified ‘whether or not genome editing of embryos constitutes *medical treatment*, given that the “patient” does not yet exist’ as a key ethical question.^39^

This section first considers the evidence taken by the HCSTC in March 2017: this reveals that ‘treatment’ is sometimes used in connection with the *future child* and sometimes the *prospective mother (or parents)*, or both. It then turns to consider similar use of the term ‘treatment’ in the leading international reports, which post-date this UK evidence session.

Turning first to the evidence taken by the HCSTC, Professor Robin Lovell-Badge, a scientist,^40^ observed: ‘You would use genome editing only where the condition you were trying *to treat* or *prevent* was a serious genetic disease.’^41^ Here, ‘treat’ or ‘prevent’ clearly refer to a condition in the future child. He later referred to a distinction drawn in the (then forthcoming) 2017 National Academies of Sciences, Engineering, and Medicine (NASEM) report, on whose committee he sat, between ‘*treating* disease or suffering and additional things like *designer babies, enhancement* or whatever’, and stressed that ‘[b]asically, we are saying that it has to be for treating or avoiding *serious disease*’.^42^ Here he seems to emphasise that HHGE will be used for what may be viewed as serious reasons. Such use of ‘treating’ appears intended to distinguish ‘treatment’ from ‘enhancement’ and ‘design’. Lovell-Badge again referred to ‘treatment’ in connection with costs, describing HHGE as ‘a one-off *treatment* that would *benefit* the individual born and perhaps their children’.^43^

‘Treatment’, juxtaposed against ‘enhancement’, was also used by Philippa Taylor, a Christian policy adviser,^44^  *opposing* legalisation of HHGE.^45^ However, she later distinguished ‘between *not creating* a child that is born with a disorder and *treating* an embryo … with that disorder’, criticising use of ‘cure’ and stressing that HHGE would ‘just *prevent* … someone with that disease from being born’.^46^ In effect, she emphasises that embryos have to be created to be edited. Significantly, Dr Andy Greenfield, a scientist,^47^ here observed: ‘I agree. The treatment is usually for the *mother*; it is the woman’s body that is the basis of much assisted reproductive technology.’^48^ Here we see recognition, also from a scientist, of the difficulty of using ‘treatment’ in relation to embryos that must be created to be edited.

James Lawford Davies, a lawyer,^49^ referred to PGT and MRTs for serious genetic conditions as closely regulated under the HFE Act 1990 (as amended), suggesting that HHGE would be ‘strictly limited to *therapeutic* use in circumstances where there is a significant risk of serious disease’.^50^ Here ‘therapeutic’ appears to refer to the future child, though this is unclear, and its use in connection with a ‘significant risk of serious disease’ again emphasises the serious reasons for using HHGE. He also noted that it would currently be illegal to use HHGE ‘in treating a *woman*’.^51^ Here he may be mindful of the term ‘treatment services’ in section 2 of the amended Act, discussed above, and also when he cites the UK as ‘a fantastic regulatory environment to do research and offer *treatment* in this area’.^52^ Nonetheless, in due course he referred to the NASEM report, for which he was a commentator, as ‘setting the bar high for research, whether it is somatic *or* germline, and potential *therapeutic* applications’,^53^ apparently not distinguishing somatic from germline with regard to the concept of ‘treatment’, and thus implying a person is treated in both cases (in one case the future child); and later he referred to ‘germline *treatment*’, which would again seem to refer to ‘treatment’ of the *future child*.^54^

Turning to the leading international reports, the idea that HHGE is a form of treatment for a *future child* can be found in the NASEM report, which considers both germline and somatic editing. With regard to the former, this recommends ‘[p]ermit[ting] clinical research trials only for compelling purposes of *treating* or *preventing* serious disease or disability, and only if there is a stringent oversight system able to limit uses to specified criteria’.^55^ The phrase, or a variation of it, ‘treating or preventing’ is repeated at various points in relation to both types of editing. The use of ‘treatment’ may reflect, in part, the Statement of Task, noted at paragraph 2 as (in part): ‘What are the potential clinical applications that may hold promise for the *treatment* of human diseases?’.^56^ As regards ‘benefit’, implicit in ‘treatment’, in the case of germline editing the report suggests this may be twofold: ‘[b]enefits from such editing would accrue to any *future child* born with reduced burden from genetically inherited disease, and to the *prospective parents* seeking to have a genetically related child without fear of passing along a disease.’^57^ Thus, while the child is deemed a beneficiary (that is, when born), so too are the parents. Logically, the notion of ‘reduced burden’ assumes that the child would otherwise have been born with a greater disease burden and I discuss the complexity of this point in Section IV.

In its 2020 report, the International Commission, of which Greenfield was a member, observes that ‘[o]ne potential alternative to HHGE for the *treatment* of genetic diseases is somatic genome editing’; it also refers to ‘assessing the balance of benefits and harms for any given *treatment* …’; and in various places, the report refers to ‘treatment’ in connection with editing zygotes.^58^ All these uses appear to refer to the future child as the beneficiary of ‘treatment’. However, later the report notes that ‘[a]s an ART, HHGE would be directed to creating a person without a specific genetic disease (as is the case with PGT), *rather than treating* an existing patient with a disease’, and alluded in the accompanying footnote to philosophical questions that were beyond the report’s scope.^59^ PGT is, of course, a form of selective reproduction. Here the report explicitly suggests it is inappropriate to use the term ‘treatment’ as regards the *future child* edited at the embryonic stage.

Taking a rather more direct approach to the issue, the Nuffield Council on Bioethics (NCOB) report of 2018 identifies the question of how to frame the issues of ‘treatment’ and ‘benefit’ early in its report. It cites from its 2016 report: ‘Genome editing is not straightforwardly therapeutic … [but] it *is* … therapeutic, in … that it potentially overcomes infertility … and it *is* preventative in that, taking the decision to reproduce as … one that a couple is entitled to make … it may prevent … [their] child … [having] a serious or life-limiting disability.’^60^ Thus, the report notes that, as regards a future child, HHGE is not ‘treatment’ but is instead ‘preventative’, while noting that it may be ‘therapeutic’ for the parents when used in relation to infertility *per se*.^61^ There are some references to HHGE as ‘treatment’ in the 2018 report, such as ‘currently unproven *treatment*’;^62^ and in Principle 1, which refers to HHGE having to be ‘consistent with the welfare of a person who may be born as a consequence of *treatment using* [gametes or embryos]’.^63^ Since this latter passage does not refer to treatment *of* gametes or embryos, it likely implies that treatment is of the *prospective parents* (particularly the woman) and should be read against the backdrop of its early framing of the issues, which have in mind the prospective parents’ possible interests in choosing to avoid the birth of an affected child.

Finally, in 2021 the WHO Committee responsible for the report on HHGE, whose membership included both Lovell-Badge and Françoise Baylis (apparently a critic of HHGE),^64^ reserves ‘treatment’ for ‘Prenatal (in utero) and postnatal somatic genome editing’, the aim of which is ‘[t]o *treat* genetic disorders’.^65^ Significantly, it refers to HHGE as a means ‘[t]o *correct* the mutant allele for monogenic disorders’.^66^ Here the aim is stated as ‘avoid[ing] inheritance of genetic disorders’.^67^ The use of ‘correct’—linked with avoidance—likely reflects a choice to avoid ‘treatment’ in relation to the future child, for reasons evident in the ethics debate, principally considered in the following section, although the term ‘correct’ is also used at times in the other reports discussed above. The 2021 finding of the EGESNT that the use of ‘treatment’ implies the presence of disease, so the term can only apply in the context of *somatic* editing,^68^ is consistent with the WHO’s approach.

As will be apparent in my analysis of the ethics debate that follows, ‘correction’ (and with it ‘avoidance’) rather than ‘treatment’ is the better term as regards the future child. With reference to various elements of the ethics literature, the following section first notes early presentation of HHGE as ‘treatment’ for a future child; next considers the challenge to this idea, typically from opponents of HHGE; then seeks to clarify the stages involved in IVF and HHGE to consider whether the term ‘treatment’ is ever appropriate in relation to the future child; and finally considers what may underlie the prevalence of the term ‘treatment’ (for the future child) in discussion of HHGE.

## IV. THE ETHICS DEBATE—THE CHALLENGE TO ‘TREATMENT’

Turning to the ethics literature, a ‘simple case’ in favour of HHGE as ‘treatment’ was offered in 2017, for example, by Christopher Gyngell and Julian Savulescu, who argued that ‘[t]here are strong *pro tanto* moral reasons to cure rare diseases … and to prevent rare genetic conditions’;^69^ that HHGE is a form of ‘‘ultimate cure’ … treat[ing] disease at its very root’;^70^ and so there is a ‘strong therapeutic case’ for HHGE.^71^ However, there has been a robust reaction to the presentation of HHGE as ‘treatment’ or ‘therapy’ for the future child either in sections of the international reports discussed above, or in the ethics literature such as this, as explored below.^72^

### A. The challenge

This reaction is evident, for example, in a 2020 ‘Geneva Statement’ on HHGE, subtitled ‘The Need for Course Correction’, with 21 co-authors including ‘public interest advocates, policy experts, bioethicists, and scientists’,^73^ amongst them Baylis: ‘[T]he most fundamental and widespread *misrepresentation*’, these authors state, is that HHGE ‘is *needed* to *treat* or *prevent* serious genetic diseases’.^74^ Rather, HHGE ‘would not *treat, cure or prevent* disease in any *existing person*’.^75^ If we take ‘existing person’ to mean a born person, this is self-evident, so the import of this statement is more apparent in what follows: namely that HHGE would ‘*modify* the genes of future children and generations through the *intentional* creation of embryos with *altered* genomes’.^76^ A similar critique is developed separately by two of these authors, Marcy Darnovsky and Katie Hasson, who suggest that HHGE ‘is *not*, by definition, *a medical matter* … it would not *save* any lives, *cure* any disease, *treat* any patient, or prevent anyone from getting sick, because it could affect only human beings who do not yet exist’.^77^ Similarly, Tina Rulli argues that ‘[g]enetic modification of gametes and embryos *does not save lives, cure diseases*, or offer a unique possibility for *disease prevention*’.^78^ These are all challenges to what has been dubbed the ‘benefit’ argument’.^79^ Thus, whether or to what extent HHGE can be described as ‘therapy’ or ‘treatment’ has become a key issue in the evolving literature.

### B. Clarifications

Assuming a decision is made to undertake HHGE prior to IVF, this would involve creating embryos by IVF in order to edit them, and this is necessarily intentional, as the Geneva Statement observes. Such creation will affect *who* may subsequently be born, determining the numerical identity of the future person(s), on Derek Parfit’s Origin View.^80^ Since these embryos, and the person or persons who would result from them following HHGE, would not otherwise have existed, it is problematic to think of benefitting a future person, other than in an existential sense: as Thomas Douglas and Katrien Devolder note, if the decision to create embryos and the editing process are viewed as ‘composite’, the ‘benefit argument clearly fails, since … editing out disease … affect[s] whether the edited … child comes into existence’.^81^ Viewed in this *composite* sense, HHGE can only be considered beneficial, *other than* in an existential sense,^82^ on a non-person affecting, or impersonal approach:^83^ that is, an approach which recognises that a person without genetic disease may be born instead of another with the disease. This approach appears to underlie the revised position of Gyngell and Savulescu, in their joint piece with Thomas Douglas. Noting Darnovsky’s critique, above, ‘[t]he medical case’ is now presented as ‘to *prevent* genetic disease’:^84^ there are ‘moral reasons to prevent the occurrence of disease in future people … [which] apply regardless of whether our … actions … make any future people *better off* than they would *otherwise have been*’;^85^ here the authors refer, by way of analogy, to the eradication of infectious disease.^86^ This again implies an impersonal understanding of ‘benefit’.

Nonetheless, we can separate the decision to create the embryos from the editing process itself, in order further to explore the ‘benefit argument’, as Douglas and Devolder do.^87^ For example, Robert Sparrow notes the distinction between ‘the history *of the events* that led to the birth of a genome-edited individual’,^88^ in relation to which the editing appears ‘*identity* affecting’,^89^ and ‘the history *of the particular embryo* that developed into a genome-edited individual’,^90^ regarding which HHGE is then ‘*person affecting*’.^91^ Likewise, César Palacios-González refers on the one hand to the ‘clinical decision to employ’ HHGE, and the ‘actual editing process’ on the other.^92^ He notes that the latter *could* be so extensive as to change the *numerical identity* of a future individual, but that otherwise it would only change a *quality* of the individual, so that an edit could be said to be person-affecting.^93^ Palacios-González also notes that the decision to employ HHGE *could* be made after the creation of a given set of embryos, so that HHGE to avoid a serious genetic condition in one of those embryos might, since the embryo already exists, be said ultimately to be person-affecting and, on his view (all going well), therapeutic.^94^ However, considering the creation of embryos as distinct from editing them (rather than in the ‘composite’ sense, as above), Douglas and Devolder have concluded, following careful consideration of various counterfactual scenarios relating to the decisions that prospective parents might make about given embryos, that, particularly in relation to serious genetic conditions, it is unlikely that an embryo will be transferred to a woman’s uterus if not successfully edited, and so the future ‘child would not otherwise have existed’, and ‘the [therapeutic] benefit argument fails’.^95^

In the light of these clarifications, the presentation of HHGE as ‘treatment’ or ‘therapy’ for a *future child* in aspects of the parliamentary evidence, policy reports and literature noted previously is problematic, at least in the absence of further explanation, as those challenging such presentations have rightly noted. It is helpful to consider what may underlie this use of ‘treatment’, and I consider this in the following section.

### C. ‘Treatment’, not ‘Selection’, ‘Enhancement’ or ‘Design’

The presentation of HHGE as ‘treatment’ for a future child may be intended to distance HHGE from selection practices such as PGT, together with concerns about ‘eugenics’, and also from the ideas of ‘enhancement’ and ‘design’.

#### 1. ‘Treatment’, not ‘selection’

First, HHGE may be presented as ‘treatment’ to distinguish it from *selection* practices. As others have noted, the latter may be thought more susceptible to concerns relating to the disability critique—for instance, the concern that such practices express the idea that people with a serious genetic condition should not be born.^96^ Unless one considers that embryos have the moral status of born people (and in law embryos, and fetuses, do not have legal personhood),^97^ whether selection practices at the embryonic stage can withstand the disability critique may partly turn on how one perceives prospective parents’ possible interests in choosing to avoid the birth of a child with a serious genetic condition. Such choices have been defended with regard to interests in well-being, autonomy, and equality for women (typically the main caregivers);^98^ and writers such as Tom Shakespeare have argued that prospective parents do not necessarily act in a discriminatory way if they wish to avoid the birth of a child with a serious genetic condition, for example in the light of their perceptions of their ability to cope with the needs of such a child.^99^ Nonetheless, a practice such as PGT does deselect a future child with such a condition.

Distinguishing HHGE from the selection practice of PGT was part of Gyngell and Savulescu’s ‘simple case’ for HHGE: while ‘[s]election prevents disease by changing who comes into existence’, HHGE ‘may provide a more desirable option’, since HHGE ‘to avoid disease … seems more analogous to curing a disease than PGD’.^100^ With reference to the disability critique, Giulia Cavaliere has referred to HHGE as potentially preferable since it is ‘pre-emptively therapeutic’.^101^ As she notes, this phrase was first coined by Anthony Wrigley, Stephen Wilkinson and John B Appleby when addressing the distinction between ‘treatment’ and ‘selection’ with reference to the differences between two types of MRT to avoid the transmission of serious mitochondrial disease: pronuclear transfer (PNT), which involves moving the nuclear material of an embryo to an enucleated one which has unaffected mitochondria, as compared with maternal spindle transfer (MST), which involves the same sort of transfer, but between eggs.^102^ In the case of PNT, the intervention occurs *after* an egg has been fertilised, and so this technique, they suggest, is ‘capable of *benefitting or harming* any child created in a straightforward ‘harm-to-interests’ sense’,^103^ and might then be viewed as ‘pre-emptively therapeutic’.^104^ In the case of MST, in contrast, the intervention occurs *before* fertilisation and the beginning of any determinate individual and so the latter is a form of *selection*, in relation to which ‘concerns about eugenics and human dignity may engage more strongly … than in the case of PNT’.^105^ The implication, they suggest, may be that prospective parents have greater reason to choose PNT, where possible.^106^

Of course, just as with HHGE, with PNT we can distinguish between the decision to create embryos so as to use PNT, which will affect ‘numerical identity’, as Palacios-González has noted,^107^ and the actual process of using PNT in relation to any given embryos, which will affect ‘qualitative identity’.^108^ We should also recall Douglas and Devolder’s point that ‘benefit’ may ultimately be existential in nature, since an embryo that has not been the subject of successful PNT will likely not be transferred to a woman’s uterus.

A further part of what may be at stake in the distinction between ‘treatment’ and ‘selection’ is that the former may be thought to address concerns that some people may have about *embryo discard*, notwithstanding that embryos must be created to be ‘treated’. The positive feature of HHGE, on this view, is that it ‘treats’ certain embryos that may otherwise be discarded.^109^ While, as noted above, the only benefit to an embryo may be existential, since it is unlikely that it would come to be a born person if not successfully edited to correct for a serious genetic condition, existential benefit may be precisely what is valued by those concerned with embryo discard.

#### 2. ‘Treatment’, not ‘enhancement’ or ‘design’

Presenting HHGE as *treatment* may also partly be intended to distinguish uses that would prevent someone being born with what may be regarded as a serious genetic condition, such as cystic fibrosis (CF), from *enhancement* uses. This does not mean that the distinction is clear-cut, or that it clearly aligns with moral permissibility and impermissibility.^110^ However, the distinction has been broadly defended, for example by Sparrow.^111^ We saw it in play, for instance, in Lovell-Badge’s emphasis on ‘*treating* disease or suffering’ on the one hand, as opposed to ‘additional things like designer babies, *enhancement* or whatever’ on the other.^112^ Similarly, the NASEM report distinguishes ‘enhancement’ uses, noting ‘there is some indication of public discomfort’ regarding ‘enhancement, whether for fear of exacerbating social inequities or of creating social pressure for people to use technologies’;^113^ and its Recommendation 6.1 states: ‘Do not proceed at this time with human genome editing for purposes other than *treatment* or prevention of *disease and disability.*’^114^ Likewise, the International Commission report, which also focuses on the avoidance of serious genetic conditions, observed that ‘the barrier to social acceptability [of enhancement] would be particularly high’.^115^

In short, the use of ‘treatment’ may seek to present HHGE as morally acceptable (albeit contentious) as compared with ‘enhancement’. In the UK, this is compatible with the written evidence, for example, of the Progress Educational Trust (PET) to the HCSTC, in which it reported on its project ‘Basic Understandings of Genome Editing’: ‘many … thought that *treatment* was morally acceptable whereas *enhancement* was morally dubious’, although ‘there was significant disagreement over how to distinguish between these two categories’.^116^ That is not to say that there may not be serious ‘enhancement’ purposes, although it is also possible that these would be construed as ‘preventive treatment’ on some views, for instance, editing to increase immunity from infectious disease, as allegedly occurred in the HHGE conducted prematurely by Dr He.^117^

‘Treatment’, as opposed to ‘enhancement’, may also be intended to highlight the *limited nature of the editing process itself*, and to stress that such HHGE would not involve *design*. Of note, a key criterion (subject to public debate) of permissible HHGE in the International Commission’s work on a ‘translational pathway’^118^ concerns the nature of the editing process: changing a variant ‘to a sequence that is common in the relevant population and that is known not to be disease-causing’.^119^ This can be understood as to ‘correct’ a sequence, a term used at various points in all the reports, as noted above.^120^ It is true that this might also be said to be a ‘modification’ or an ‘alteration’, and the HFE Act 1990 (as amended) itself refers to the latter concept in relation to embryos and gametes that are not of the ‘permitted’ kind.^121^ Yet a different meaning of such terms appears intended in the Geneva Statement’s point that HHGE would ‘*modify* the genes of future children’, creating ‘embryos with *altered* genomes’, making it ‘categorically distinct’ from somatic editing.^122^ There is no ‘categorical’ difference, however, in the nature of the editing process itself, only in the question of whether this amounts to ‘treatment’, as addressed above. In this light, while Darnovsky and Hasson are broadly right to observe that somatic editing ‘treats patients’ and HHGE ‘alters embryos’,^123^ what they omit here is that gene editing *corrects* sequences in both cases. Thus, both the *intention* of the editing (to remove a variant that would be responsible for a serious genetic condition) and its *scope* (limited to correction to a common ‘naturally occurring’ sequence) are relevant to our understanding of the senses, in particular the *scope*, of ‘alter’ and ‘modify’ in play as regards HHGE.

The Geneva Statement also refers to ‘satisfy[ing] *parental desires* for … children with *specific genetic traits*’.^124^ Such use, coupled with ‘alter’ and ‘modify’, may hint at the sort of concern expressed in the US President’s Council on Bioethics Report in 2004, regarding a possible child who may be ‘*designed* to certain specifications [and] … viewed as … an *artifact*’.^125^ Yet ‘trait’ is a misleading portrayal of a genetic sequence that will not give rise to a serious genetic condition, and a desire for a child with such a sequence is different from one, say, for a child who will have the trait of being particularly tall.

Significantly, I suggest that emphasis, in policy materials, on the contrast between ‘treatment’ (aligned with ‘serious disease’, ‘disability’ and sometimes ‘suffering’) on the one hand, and ‘enhancement’ (often aligned with ‘designer babies’ or ‘design’) on the other, may be intended to emphasise a *seriousness of purpose*: in this way, ‘treatment’ may best be understood as *a proxy* for that purpose. Indeed, as the International Commission report notes, the seriousness of the genetic condition with which a child would otherwise be born is currently a central consideration for access to other ARTs that aim to avoid the birth of a child with such a condition, namely in relation to PGT (by testing and selection) and MRTs (by modification): the report refers here to ‘high’ mortality or ‘severe’ morbidity together with the idea of a lack of ‘alternatives’ for prospective parents.^126^ An emphasis on serious purposes may also be an implicit riposte to concerns about *risks* in HHGE—the risk/benefit calculus, explored further in Section VI.^127^ However, it is misleading to say that HHGE *treats* a future child, given that the only benefit of correcting for a serious genetic condition will be existential (on a ‘composite’ understanding, as discussed above).

Aligned with the notion of ‘serious purpose’ may be the concept, implicit or otherwise, of ‘need’: the idea that HHGE is ‘needed’ to avoid the birth of children with serious genetic conditions. This is highly contested, since it raises the question of what the alternatives are, and who should judge their acceptability.

## V. PROSPECTIVE PARENTS’ INTERESTS

### A. The ethics and policy debate

One way to avoid the birth of children affected by a serious genetic condition would be for prospective parents not to create them. A central strand of Rulli’s argument, for example, is that the creation of a person with, say, a serious genetic condition is *not* inevitable if HHGE is not used, since prospective parents have the choice of not procreating at all.^128^ On her view, this is the relevant ‘counterfactual condition’.^129^ Accordingly, on her view HHGE ‘does not prevent a disease that someone would otherwise have’,^130^ meaning that it does not offer a ‘unique opportunity’ to avoid it.^131^ Compatible with the idea of a lack of a need for HHGE, the Geneva Statement observes that prospective parents ‘already have several options … should they find them acceptable’,^132^ such as donor gametes, or adoption. However, it does not further consider the question of acceptability. Rulli likewise notes the availability of these options, though she also includes PGT, which would not assist where *all* of a couple’s embryos are affected.^133^

The question of ‘options’ or ‘alternatives’ has been considered in each of the leading policy reports. The NASEM report recommends that ‘the absence of reasonable alternatives’ should be one criterion in any ‘regulatory framework’ for clinical trials, together with the prevention of ‘a serious disease or condition’, amongst others.^134^ It observes that the latter terms are ‘necessarily vague’ and may be interpreted variously in different societies, while noting the US Food and Drug Administration’s (FDA) definition of seriousness.^135^ As regards ‘reasonable alternatives’, people differ in their views on this matter, as the NASEM report itself notes. For example, it seems from Rulli’s critique,^136^ and that of the Geneva statement,^137^ that various alternatives are *assumed* to be reasonable by these authors. Yet this may not be how various prospective parents view this matter.

Turning to the discussion of ‘alternatives’ in the leading policy reports, the NASEM report notes that these ‘include deciding not to have children; adopting a baby; or using donated embryos, eggs, or sperm’.^138^ However, it notes that these options mean that *both* parents will not be genetically related to their offspring, something of ‘great importance to many people’.^139^ Having discussed scenarios in which all or most of a couple’s embryos would be affected,^140^ the report observes that while the number of affected couples may be ‘small’, prospective parents’ ‘concerns … are real’,^141^ concluding that sometimes HHGE ‘might be the *only* or *most acceptable option*’ where such parents seek an unaffected genetically related child.^142^ In this way, the report gives considerable weight to *prospective parents’* possible evaluations of the reasonableness of the options open to them.

The NCOB report notes that prospective parents may desire to have not just *a* child, but one who is *both* genetically related to them *and* who does not have a serious genetic condition.^143^ As regards ‘alternative reproductive options’, their equivalence ‘will depend on … assumptions, understandings and values that can be argued out in different ways’.^144^ Regarding the desire for genetically related children, the report observes that ‘[w]e may … have good reasons to respect … the desires of people for whom we should, a priori, have respect’;^145^ and that ‘it is axiomatic’ that such respect entails that prospective parents can freely choose ‘how they pursue their reproductive project’, but that such choice is not ‘practically significant’ in the absence of ‘*acceptable options*’.^146^

The International Commission report is both focused and explicit, observing that ‘[a] key consideration is whether the prospective parents already have *reasonable options for conceiving a genetically-related child*’ without a serious genetic condition.^147^ A lack of such options, or ‘extremely poor’ ones,^148^ together with the seriousness of the genetic condition in the future child, form part of the core of the report’s recommendations for possible initial uses of HHGE, considered in further detail in Section VI. This approach is consistent with that of the NASEM report (noted above), though it seems to go further in explicitly focusing on ‘reasonable options’ to have an *unaffected genetically related child*, as compared with the NASEM’s comparatively broader reference to ‘the absence of reasonable alternatives’.

As can be seen, these policy reports each appear to regard the desire for genetically related (unaffected) children as one that should be respected, if possible, and to take the view that the acceptability of the alternatives is a judgment for prospective parents to make.

Indeed, turning to the UK, there are various features of the alternatives frequently cited by opponents of HHGE that such parents may wish to consider. The option of adoption is not straightforward, entailing: complex assessment processes for prospective adopters and ongoing visits for some time post-adoption;^149^ the possibility of having to facilitate ongoing contact with a child’s birth family, if this is considered in her or his best interests;^150^ and potential challenges as regards bonding, particularly since most UK children needing adoption have suffered abuse or neglect.^151^ As for donor conception, although empirical evidence shows donor-conceived families are doing well,^152^ the option entails a legal requirement that prospective parents be advised, prior to treatment, that it is thought best to tell a subsequently born child about her/his donor conception;^153^ and direct-to-consumer testing may result in changes to the current law under which a donor remains anonymous until a child is 18,^154^ potentially making child-raising and family creation more complex for parents.^155^ In this light, as I have argued elsewhere in more detail,^156^ I suggest that it is for *prospective parents*, who may greatly value the prospect of a genetically related child, to judge the acceptability of alternatives, and I reject what appear to be assumptions about the reasonableness of alternatives made by the authors cited at the start of this section.

I now consider the way in which a sympathetic approach to many prospective parents’ desire for unaffected genetically related children, where they have no or poor options to achieve this without HHGE, might be developed in law.

### B. The law

As regards UK law, following a process of ‘broad and inclusive societal debate’ as recommended by the NCOB,^157^ Parliament could amend various sections of the HFE Act 1990 (as amended) to legalise HHGE, with HHGE understood as a ‘treatment service’ in the manner that PGT and MRTs are. HHGE would thus be ‘treatment’ for the prospective parents, particularly the woman, under the amended Act, enabling couples for whom there are no, or poor options to do so, to have an unaffected genetically related child. Possible clinical scenarios are discussed in Section VI.

More generally, this change could be supported, in part, with reference to wider common law: prospective parents’ common desire for genetically related children has been recognised, for instance, by the Singapore Court of Appeal in *ACB v Thompson*,^158^ and arguments in favour of legalisation could also be developed with reference to this case, which highlights the ‘ordinary’ nature of genetic relatedness between parents and children, and the ‘deep desire’ that many prospective parents have for such relationships.^159^

As regards relevant human rights law in support of this approach, the right to have (or not to have) a genetically related child was recognised by the European Court of Human Rights (ECtHR) with reference to Article 8 in *Evans v United Kingdom (Evans)*;^160^ and the interest in being able to choose whether to have an unaffected child with the aid of PGT was recognised in *Costa and Pavan v Italy.*^161^ It could therefore be argued that prospective parents have a right, under Article 8, to use HHGE to have a genetically related child without a serious genetic condition.^162^ While the ECtHR may at some point make this finding in a challenge to non-legalisation of HHGE in any given Convention State, such a right would nonetheless be subject to the limiting provisions of Article 8(2), particularly relating to ‘health’ and ‘morals’. In this regard, the question of the necessity (or otherwise) of interfering under Article 8(2) to justify non-legalisation of HHGE would be assessed with reference to the criteria in *Handyside v United Kingdom*: whether there is a ‘pressing social need’, with the interference being ‘proportionate to the legitimate aim pursued’ and justified with reference to ‘relevant and sufficient’ reasons.^163^ Although rigorous scientific appraisal of the safety of HHGE should allay objections regarding ‘health’, as regards ‘morals’ arguments would likely have to assuage concerns about ‘identity’ and ‘dignity’, such as those at stake in the international conventions noted earlier. While some will take the view that this should not be a significant hurdle in the case of the avoidance of serious genetic conditions (recall the UK government’s decision that MRTs are not contrary to ‘dignity’ in Article 24 of the UNHGHR), not all will agree, and the Court is likely to be mindful of the margin of appreciation it gives to Contracting States in relation to sensitive ethical issues.

This may well work in favour of objections to the legalisation of HHGE in conservative ECHR States, as evidenced by the problematic decision of the ECtHR in *SH and Others v Austria (SH)*.^164^ In this case, which concerned access to donor gametes in the course of IVF, while the Grand Chamber recognised ‘the right of a couple to conceive a child and to make use of medically assisted procreation for that purpose’ under Article 8,^165^ and reiterated its statement in *Evans* that ‘[w]here a particularly important facet of an individual’s existence or identity is at stake, the margin allowed to the State will normally be restricted’,^166^ it purported to rely on the Court’s consensus doctrine to hold that the margin of appreciation remained wide;^167^ it therefore found no breach of Article 8.^168^ However, its analysis of the legal state of play amongst Convention States was flawed: it wrongly held that there was no consensus at the time the applicants began their legal action, referring to at best an ‘emerging consensus’ since that time.^169^Accordingly, the margin of appreciation had in fact been reduced.

A second aspect of the decision in *SH* concerned the Grand Chamber’s deference to the highly conservative approach of the Austrian government: it reasoned, for example, that the use of donor gametes with IVF entails a ‘highly technical medical process’, one which must consider ‘human dignity’ as well as the ‘wellbeing’ of donor-conceived children.^170^ This is notwithstanding that the Convention itself makes no reference to ‘dignity’, as others have noted.^171^ This is the kind of vague, constraining use of ‘dignity’ critiqued by Caulfield and Brownsword with reference to the international instruments discussed earlier.^172^ The Grand Chamber also highlighted concerns about ‘selection’ of children which were unconvincing in the context under consideration.^173^ Taken together, the Grand Chamber’s use of the consensus doctrine and reliance on the Austrian government’s arguments meant that it never properly addressed the supposed necessity, under Article 8(2), of the interference in the applicants’ Article 8 rights.

Returning to HHGE itself, if its non-legalisation to avoid a serious genetic condition in a future child in a given Convention State were challenged in the ECtHR, opponents’ arguments that non-legalisation is necessary under Article 8(2) would likely refer, in part, to the constraining sense of ‘dignity’ just noted, perhaps particularly in relation to embryos, notwithstanding that, as noted earlier, the Court has not held that these have a right to life under Article 2.^174^ Indeed, the idea that the embryo does not have such a right (or can *at best* have a highly limited one) is a necessary implication of the consensus, amongst Convention States, that a woman can terminate a pregnancy for reasons relating both to her life *and* health, albeit (and problematically so) not one recognised by the Court itself.^175^ While there is some reference in *Vo v France* to embryos in connection with ‘dignity’,^176^ HHGE to avoid a serious genetic condition is not plausibly an affront to any ‘dignity’ that the embryo might be thought to have; in contrast, it could perhaps be argued that HHGE for trivial aesthetic reasons (unlikely to be possible) might be.

However, even if the Court were to adopt a more autonomy-oriented approach to ‘dignity’ (in relation to Article 8 arguments) than in *SH*, perhaps mindful of its relatively recent finding in *Parillo* v *Italy* that reproduction is a ‘core right’,^177^ the consensus doctrine could be deployed to lead to a finding of no breach of Article 8 in a Convention State not willing to legalise HHGE, at least for some years: HHGE will be entirely novel, if ever legalised, and it would take time for a consensus to form in favour of legalisation. Where non-legalisation reflects a national process of public consultation and democratic decision-making, this may be defensible. However, both George Letsas and Eyal Benvenisti have expressed concerns about the way the moral views of the majority may impact on the Court’s use of the margin of appreciation and consensus doctrines.^178^ Much will turn on the quality of the arguments made: as the Grand Chamber’s support for the Austrian government’s arguments in *SH* shows, and as was noted earlier, the contested notion of ‘dignity’ is not a firm footing for policy and law in relation to new technologies.^179^ Unsurprisingly then, although the WHO referred to ‘dignity’ in its draft report on HHGE (and somatic editing),^180^ it dropped all reference to it in its final report.^181^ As for arguments relating to the welfare of the child (used against the applicants in *SH*), these have the potential to be very strong in relation to the avoidance of a serious genetic condition in a couple’s child or children, and thus to work in favour of legalisation, providing concerns about the risks of editing have been addressed, though arguments should acknowledge that the ‘driver’ for HHGE is prospective parents’ desire for unaffected genetically related children, not ‘treatment’ of a child, since an embryo must be created to be edited.

The discussion so far has established that, as a matter of ethics and law, HHGE is best understood as treating the *prospective parents*, not the future child. As Peter FR Mills has noted, its purpose would be ‘to enable choices about what kinds of people may exist’.^182^ Where prospective parents have a desire for an unaffected genetically related child, the use of HHGE would aim to secure this by avoiding the transmission of a serious genetic condition that would, or may, otherwise occur if they decided to try to have a child without such assistance, in ways explored in detail below. Furthermore, HHGE could be said to be ‘needed’, in the instrumental sense,^183^ to support them in their desire to have unaffected genetically related children, at least in the sorts of cases now considered.

## VI. HHGE IN CLINICAL PRACTICE

To what extent clinical practice should support prospective parents at risk of having a child with a serious genetic condition is considered in this section with reference to clinical scenarios discussed in the International Commission report.^184^ The analysis also provides the opportunity to consider whether, as regards the disability critique, HHGE has any moral advantages over the selection practice of PGT.

### A. All of a couple’s embryos affected

Where all of a couple’s genetically related children would inherit a ‘serious monogenic’ disease, this would fall into the report’s ‘Category A’.^185^ Examples include ‘autosomal dominant diseases such as Huntington’s disease; and autosomal recessive diseases such as CF, sickle cell anemia, and beta-thalassemia’.^186^ The number potentially affected for several different diseases^187^ would be ‘very rare’.^188^ For instance, for CF there may be ‘several such couples’ in Europe, though improved treatment (of people living with CF) may increase the numbers.^189^

Understandably, in this light critics have stressed that in ‘nearly every case’, as the Geneva Statement notes, prospective parents could use PGT.^190^ Rulli suggests that PGT ‘is just as successful’, which is not accurate and is contradicted by her own statement that ‘[o]nly in the rarest of cases would … [HHGE] offer unique value’.^191^ Whether HHGE should be legalised, suggest Darnovsky and Hasson, ‘turns not on preventing the births of children with inheritable disease, but on providing genetically related children to those few people for whom … [PGT] would not work’.^192^ Given the possible numbers at stake in Category A, there is some point to these criticisms. Nonetheless, the small numbers can be seen more positively: the report notes that ‘it would be suitably cautious to begin with a small number of couples who have no alternatives, proceed carefully, and intensively study the result’.^193^ That said, the small numbers do not themselves speak to the justification of use in Category A.

As regards the report itself, questions about the justification for HHGE, and acceptable conditions of its use, are apparently answered with reference to the four criteria in its Recommendation 4,^194^ namely: *first*, children would otherwise inherit serious monogenic diseases; *second*, editing would change ‘a pathogenic variant known to be responsible for the … disease to a sequence commonly carried in the relevant population’; *third*, no subsequently born ‘individuals resulting from edited embryos could have been exposed to potential harms from HHGE without potential benefit’, since all the embryos of a given couple in Category A carry the relevant genotype; and, *fourth*, in Category A, HHGE is the ‘only’ option for couples who wish to have a genetically related child without the disease in question (the alternative limb of this criterion being whether a couple’s options for doing so are ‘extremely poor’, considered in relation to Category B, below).^195^

Three of these four criteria have been discussed at various stages in earlier sections: seriousness, the intent and scope of the editing, and alternatives. Here I focus particularly on the third: how we should make sense of the requirement that if someone is born as a result of embryo editing, followed by a woman’s pregnancy, there should have been no risk of potential harm without potential benefit from the editing.

The difficulty with reflecting on the alternative scenario in which the person is born without the editing is that, as explored by Douglas and Devolder (as discussed earlier), it is most unlikely that the prospective parents would elect to transfer an embryo that had *not* been successfully edited and would thus result in a child with a serious genetic condition, and so the ‘unedited child’ (with whom this criterion requires implicit comparison) will almost certainly not come to exist.^196^ This represents a challenge to the notion of ‘benefit’ in the report’s third criterion, unless this is simply understood in an existential sense.

A further question is whether the transfer of an unedited affected embryo would be permitted under the HFE Act 1990 (as amended), should HHGE be legalised by further amendment to the Act. In the case of PGT, for example, the Act states that affected embryos ‘must not be preferred’; and the Human Fertilisation and Embryology Authority’s (HFEA) *Code of Practice* states that this ‘applies only where there is at least one other embryo suitable for transfer that is not known to have the characteristics’.^197^ This kind of provision would be irrelevant to HHGE for Category A because *all* of a couple’s embryos are affected. However, as regards PGT, the *Code* also states that ‘[t]he use of an embryo known to have an abnormality … should be subject to consideration of the welfare of any resulting child and should normally have approval from a clinical ethics committee’.^198^ This is consistent with the current obligation to consider the welfare of the child under section 13(5) of the amended Act,^199^ and such a provision could be written into the *Code* in relation to HHGE. Although section 13(5) has rightly been critiqued in relation to IVF generally, notably by Emily Jackson,^200^ a concern with the welfare of the future child—expressed through *some* form of regulatory oversight—may have at least some validity in relation to the question of whether an affected embryo should be transferred to a woman’s uterus in the context of PGT or HHGE, subject to recognition of the important caveat that, as long as a future child would have a life of overall positive value (one that s/he would think worth living), it would not be contrary to her/his interests to transfer the relevant embryo, since that child would have just one chance of life.^201^

Notwithstanding the need for some caution in applying a welfare assessment prior to transfer of an affected embryo, returning to the report’s third criterion, the idea that any potential *risks* of editing (here I avoid ‘harm’, which is comparative) on the one hand, should be balanced by the removal of a serious genetic condition on the other, is comprehensible, and may be one that at least some people would support, though the public should be consulted about this matter.

While a child born following HHGE could not be benefitted, other than in an existential sense (that is, where the decision to create embryos and edit them are viewed as ‘composite’, as discussed above), Category A presents a strong case for the use of HHGE since *every* embryo that a couple could produce that is genetically related to them would be affected, and so there is no alternative way for them to have a genetically related child if they very much want this. The question of alternatives is, as noted above, the report’s fourth criterion and, as noted earlier, apparently likewise an important criterion in the NASEM report, and a clear indicator for HHGE in the NCOB report. Moreover, given these prospective parents’ wishes, they will benefit from being enabled to (try to) have a genetically related unaffected child and IVF and HHGE would be, as concluded earlier, a ‘treatment’ for them.

#### 1. Testing and selection in Category A cases

Given the emphasis in the debate in favour or against HHGE on the distinction between *treatment* and *selection*, we should also consider the ways in which the processes associated with HHGE may include elements of selection, as Sparrow and Robert Ranisch have both emphasised, together with critics of HHGE.^202^

For Category A embryos, the report’s Recommendation 6 is that all embryos should be biopsied at the blastocyst stage to demonstrate ‘the existence of the intended edit in all biopsied cells and no evidence of unintended edits at the target locus …[or] of additional variants introduced by the editing process at off-target sites’.^203^ In theory, this could result in one or more embryos being discarded (deselected) to avoid risks to a future person. Although someone cannot complain about being born unless s/he thinks that her/his life is not worth living,^204^ very few people are likely to support a regulatory regime, as regards a new technology such as HHGE, that does not take a cautious approach to the health and well-being of the future person in the way encompassed in Recommendation 6. Such an approach is inherent, not only in the International Commission report, which takes its lead from a 2016 US report on MRTs in making the welfare of the future child the ‘primary value’,^205^ but also in Principle 1 of the NCOB report, which states that embryos ‘should be used only where the procedure is carried out in a manner and for a purpose that is intended to *secure the welfare of* and is *consistent with the welfare of a person*’.^206^ While this need not imply that the future person is being ‘treated’, it may do so in the absence of further explanation, for instance given the use of ‘purpose’.

Beyond PGT to assess the safety and efficacy of the HHGE, embryologists will select the embryo/s with the best chance(s) of resulting in a live birth. If one assumes, for simplicity, that those deselected at this point would not come to term if transferred, then this is not ‘selection’ in any substantive sense, such as that normally associated with reproductive technologies to avoid a serious genetic condition, such as PGT. Furthermore, not to choose those with good potential for live birth would be harmful to the woman and her partner since, even if a pregnancy were established, it would likely fail. This would be clinically counter-productive. Of those embryos (if any) with good potential for live birth, one (or more under some circumstances) may be transferred to a woman’s uterus,^207^ with her consent, and others frozen with the intention that they may be thawed and transferred for a subsequent pregnancy.

In sum, the *selection* processes that would be at stake in the use of HHGE in Category A are not the same as those in PGT. In the latter case, they are directed to avoiding the birth of a future person with a serious genetic condition in favour of another without it. In contrast, selection in the case of HHGE would be concerned either to avoid uncertain risk to a future person (from the editing) or to try to give the existential good of birth to a person (choosing an embryo with good potential). In both cases, and most obviously the latter, this will of course be to benefit the prospective parents, or, put another way, to treat them.

Of note, if the alternative in a Category A case would have been that the prospective parents chose to have no child at all, then the procedures entailed in HHGE would not be selective at all. In itself, such a choice could, paradoxically and unfairly given the personal losses at stake for the prospective parents, be more susceptible to the disability critique—the idea being that such parents simply avoided giving birth to a child with the condition in question and so that child never came into existence: not so much ‘deselection’ as ‘prevention’. To use the language of the Geneva Statement it could, uncharitably, be said that they chose not to ‘welcome’ a child with a serious genetic condition into the world.^208^ Yet this implication is not addressed by those, such as Rulli, who argue that prospective parents have the option of simply foregoing having a genetically related (and seriously affected) child.

### B. Most of a couple’s embryos affected

Category B again concerns ‘[s]erious monogenic disease, with high penetrance’.^209^ The report estimates that such disease affects 0.1 percent of all couples, or approximately one million globally.^210^ While in various cases the number of affected embryos would be 50 percent and in others 25 percent, the particular concern is with the ‘rare circumstances’ with higher affected numbers: for example, ‘[i]f both parents are heterozygous for a disease-causing allele for an autosomal dominant disease, on average 75 percent’ would be affected.^211^ In this case, the initial estimate is of one couple per PGT clinic each year, compared with 50–100 for couples whose embryos would have a 50 percent chance of being affected.^212^

To meet the *fourth criterion* relating to the existence or quality of alternatives, the report considers that the number of *unaffected* embryos should be 25 percent or less, *and* that a couple should have undergone a previous unsuccessful cycle of IVF and PGT, with the implication that the couple’s options for having a genetically related unaffected child are ‘extremely poor’.^213^

There are several issues that may affect the chances of a successful outcome from PGT in this group: there may be few high-quality embryos, with no unaffected ones identified, or all identified as unaffected may be of poor quality,^214^ even to the extent that there is no viable unaffected embryo;^215^ an embryo may also be damaged by the biopsy process, making it ‘unusable’, or lowering the chance that a live birth will follow transfer.^216^ Although the couple can choose to undergo IVF and PGT again, sometimes no live birth follows ‘even after several cycles’.^217^

The burdens of such failed cycles and repetition are notable. For the woman, this will entail repeating the process of ovarian stimulation and egg retrieval. This is particularly problematic for women with an increased risk of ovarian hyperstimulation syndrome, and also for the likely larger category of women whose fertility is declining due to age.^218^ There are many reasons why a woman may be in the latter stages of her ability to reproduce: for example, she may not have met her partner until quite late; she may have deferred family creation for education or work; or she may have had health issues. Alternatively, and possibly in conjunction with one or more of the above, she and her partner may have already had a significant reproductive history before they reach the possibility of PGT, involving issues that have been discussed in relevant policy materials, such as: fertility problems for which they sought IVF; one or more miscarriages; one or more pregnancies in which the fetus tested positive for a serious condition and which the couple decided to terminate; and/or one or more births of affected children.^219^ If the physical and emotional burdens of any one of these possible histories and experiences are to be taken seriously, particularly as they affect women—who themselves undergo IVF and try to carry a pregnancy—then there is a good case in support of the report’s proposals for initial uses of HHGE also in a small subset of Category B to try to increase the numbers of high-quality unaffected embryos (although it is unknown at this stage whether HHGE would affect embryo quality).^220^ Whether expansion could be considered in due course, to reduce the burdens of IVF and PGT for couples with fewer numbers of affected embryos, would turn on issues of safety and efficacy.^221^

#### 1. Testing and selection in Category B cases

Returning to the report’s criteria for HHGE, ‘as currently conceived’ HHGE would involve submitting all zygotes to editing,^222^ and so the *third criterion* of no ‘needless’ editing would not be met unless techniques were developed, such as polar-body genotyping in some cases,^223^ to establish which zygotes are affected.^224^ If this were possible, only *affected* zygotes would be edited and PGT would then be used to check that process; and *unaffected* zygotes would be subjected to PGT when developed to the appropriate embryonic stage to confirm the earlier testing process.^225^

In short, the *selection* issues at stake in Category B are more complex than in Category A. First, in Category B cases, to avoid the issue of potentially ‘needless’ editing, there would be the attempt to establish the genotype of a zygote prior to any HHGE. However, this would not *deselect* affected embryos, as would normally be the case with PGT: rather, the attempt would then be made to correct, by HHGE, the variant that would cause a serious genetic condition. These embryos would then be biopsied at the blastocyst stage (the post-HHGE check) and those with good potential for live birth selected for possible transfer. Of the *unaffected* embryos, apart from the use of PGT, at the appropriate stage, to confirm that they were unaffected, again those with good potential for live birth would be chosen. Overall, as with Category A, the selection processes at stake in Category B would either be concerned with the health and well-being of the future child or would aim to confer an existential benefit or good and, most obviously in the latter case, this would treat the parents (as does standard PGT, where sought).

### C. HHGE, correction, selection and treatment

The discussion has established that in both the proposed initial categories—Category A and a small subset of Category B (but applicable to *any* use in Category B)—testing and selection in relation to a serious genetic condition would not result in embryo discard on *this basis*. The practice of HHGE would therefore not be subject to the disability critique of testing and selection practices, unless the aim of correction is thought problematic in itself. This takes the sting out of the criticism that HHGE will likewise involve selection techniques and that, from the perspective of the disability critique, it has no moral advantages over a technique such as PGT.^226^ Moreover, HHGE will not have been ‘at cost’ to any embryos (post-creation, that is), since none will have been deselected on the grounds of a serious genetic condition, and such selection as occurs will have been to guard against risks from HHGE to the future child that an embryo could become, or to confer an existential benefit.

The decision to use IVF together with HHGE, followed by a successful pregnancy, *will* affect the numerical identity of a future child, given that this will involve a different child coming into existence than one who may otherwise be born to a couple. If this means that this process is another form of ‘selective reproduction’—for instance because a Category A couple would instead have created a child with donor gametes, or because a Category B couple would have persisted with cycles of PGT—then it should be said that this is not the same sense of ‘selection’ as that at stake in PGT by itself. Moreover, as regards *prospective parents’* interests, the use of HHGE in Category B would be morally advantageous as compared with PGT, to reduce the emotional and physical burdens of repeated cycles of IVF and PGT alone. Both in Category A and Category B, the parents can be said to be offered treatment and, if IVF, HHGE, and pregnancy are all successful, treatment will have been efficacious.

## VII. CONCLUSIONS

HHGE is best explained, and *morally justified*, with reference to the common desire of prospective parents’ (and likely their descendants too) for genetically related, and unaffected, children. For those prospective parents whose embryos would all be affected (Category A) or mostly affected (a subset of Category B), and potentially also for those for whom half or fewer would be affected (namely, others in Category B), a successful process of IVF and HHGE, with relevant selection processes, followed by a pregnancy that goes to term and results in a live birth, would treat such prospective parents. Most strikingly in Category A cases, this could also be said to ‘prevent’ the birth of children with serious genetic conditions, since every embryo couples in this category could produce would carry the relevant genetic condition, and given the acceptance, as argued for here, of prospective parents’ desire for a genetically related unaffected child coupled with the possibility, or even likelihood, that such parents would otherwise try to have a child without assistance. Such treatment would be particularly beneficial for women, on whom principally, and by a long way, fall the physical burdens of child creation. The emotional losses of reproductive failure fall on both men and women but may particularly negatively affect women.^227^ While the idea of treatment for *prospective parents* is implicit, to a greater or lesser degree, in the international reports discussed here, this should be made much clearer in policy debate.

At the same time, as regards the *future child*, it is right to focus on HHGE being used in a way that is compatible with her or his health and well-being, since this speaks to the appropriate conditions of use of HHGE, and hence the *moral permissibility* of certain instances of HHGE to treat prospective parents. When the International Commission and NCOB reports highlight the welfare of the future child in the conduct of HHGE, this rightly guards against risks to a future child but should not be understood as a concern with treating that child, who can only benefit in an existential sense (that is, where the decision to create embryos and edit them are viewed as ‘composite’, as discussed above). Thus, the use of ‘treatment’ in relation to a future child should be avoided, as the authors of these reports likely recognise. Viewed charitably, such use in policy materials and broader debate may partly be intended to convey *a seriousness of purpose*, particularly to distinguish HHGE for serious genetic conditions from *enhancement* purposes (not necessarily definitionally clear-cut but generally suggestive of lesser gravity): ‘treatment’ may thus be *a proxy for seriousness of purpose.*

The term may also be used to distinguish HHGE from *selection* against genetic conditions by means of PGT. As we have seen (in discussion of Categories A and B), while the conduct of HHGE *would* involve instances of testing and selection, this would almost certainly not entail embryo discard on the grounds of a serious genetic condition. Accordingly, there does appear a moral difference between HHGE and standard PGT, making the former less susceptible to the disability critique of selection practices, and notwithstanding that a future child is not ‘treated’ by HHGE, as outlined above.

As regards terminology, ‘correction’, as can be found particularly in the WHO report, though also in the other reports, is the better term. This is also preferable to ‘modify’ and ‘alter’ which, without further explanation, or combined with terms such as ‘traits’, may have, and be *intended* to have, implicit (and, problematically, not necessarily defended) moral connotations of inappropriate interference or ‘design’. Since the public has an important role in debates regarding the legalisation of HHGE, the broadly inclusive consultation processes that will precede any attempt to legalise HHGE may benefit from such clarifications.

On the arguments considered here, and noting the further issues beyond my current scope, some of which I have discussed elsewhere,^228^ HHGE to correct for a serious genetic condition in a future child would be morally justifiable to treat prospective parents in the ways discussed, and morally permissible if conducted in a way that does not, following rigorous scientific research, knowingly create risks for the health and well-being of a future child, whom it can only be said to ‘benefit’ in an existential sense (on a ‘composite’ understanding of embryo creation and editing).^229^ Legally, it is arguable that such use of HHGE would be compatible with the international instruments discussed earlier. It would also be supportable with reference to prospective parents’ interests under Article 8 of the ECHR, although the operation of the Court’s margin of appreciation and consensus doctrines may, and for some time, impact arguments in any ECtHR challenge to the non-legalisation of HHGE in a given Convention State. As regards UK law, notwithstanding that the HFE Act 1990 (as amended) would need amendment to permit HHGE, these conclusions are compatible with the UK’s existing approach to the regulation of PGT and MRTs,^230^ together with IVF, as ‘treatment services’ to assist women to carry children, and the careful confinement of current practices to certain degrees of risk and seriousness in relation to genetic conditions, including in ways that are attentive to prospective parents’ reproductive histories, views and experiences.^231^

## Footnotes

1 National Academies of Sciences, Engineering, and Medicine, *Second International Summit on Human Genome Editing: Continuing the Global Discussion: Proceedings of a Workshopin* [sic] *Brief* (The National Academies Press 2019) 2–3.

2 Statement by the Organizing Committee of the Second International Summit on Human Genome Editing, 29 November 2018, in ibid 7–8, Box 1.

3 Eric Lander and others, ‘Adopt a Moratorium on Heritable Genome Editing’ (2019) 567 Nature 165, 168.

4 See eg Tina Rulli, ‘Reproductive CRISPR Does Not Cure Disease’ (2019) 33 Bioethics 1072 and other literature discussed in Sections IV and V. An additional ‘selection’ practice is prenatal diagnosis (PND), which involves prenatal testing of various kinds, potentially followed by selective termination, if this is what prospective parents choose, and if in accordance with s 1(1)(d) and sometimes s 1(1)(a) of the Abortion Act 1967 (as amended by the Human Fertilisation and Embryology (HFE) Act 1990).

5 See WHO Expert Advisory Committee on Developing Global Standards for Governance and Oversight of Human Genome Editing, *Human Genome Editing: A Framework for Governance* (World Health Organization 2021) (the WHO report), eg ‘Box 3. Current, potential and speculative human genome editing research’ and use of ‘correct’ and ‘correcting’ therein.

6 See eg Nuffield Council on Bioethics (NCOB), *Genome Editing and Human Reproduction*: *Social and Ethical Issues* (NCOB 2018) (the NCOB report); National Academies of Sciences, Engineering, and Medicine, *Human Genome Editing: Science, Ethics, and Governance* (The National Academies Press 2017) (the NASEM report); and National Academy of Medicine, National Academy of Sciences, and the Royal Society, *Heritable Human Genome Editing* (The National Academies Press 2020) (the International Commission report).

7 Regarding the complexity of the science, see the International Commission report (n 6). See the NCOB report (n 6) regarding the broader debate.

8 Deryck Beyleveld and Roger Brownsword, ‘Human Dignity, Human Rights, and Human Genetics’ (1998) 61 Mod Law Rev 661, 664.

9 Emphasis added.

10 Timothy Caulfield and Roger Brownsword, ‘Human Dignity: a Guide to Policy Making in the Biotechnology Era?’ (2006) 7 Nature 72, 72.

11 ibid.

12 Deutscher Ethikrat, *Intervening in the Human Germline: Opinion—Executive Summary and Recommendations* (Berlin 2018) [41].

13 Department of Health (DH), *Mitochondrial Donation: A Consultation on Draft Regulations to Permit the Use of New Treatment Techniques to Prevent the Transmission of a Serious Mitochondrial Disease from Mother to Child* (February 2014) [1.29].

14 This has 35 Member State signatories and 29 ratifications.

15 Emphases added.

16 Steering Committee for Human Rights in the Fields of Biomedicine and Health (CDBIO), ‘Re-examination Process of Article 13 of the Oviedo Convention: Conclusions and Clarifications’ (27 September 2022) at https://rm.coe.int/cdbio-2022-7-final-clarifications-er-art-13-e-2777-5174-4006-1/1680a87953 (last accessed 25 February 2024) at 3 and 4 respectively.

17 For a contrary view see Merel M Spaander, ‘The European Court of Human Rights and the Emergence of Human Germline Genome Editing – “The Right to Life” and “the Right to (Artificial) Procreation”’ (2022) 29 Eur J Health Law 458, 478, who suggests Art 13 ‘explicitly prohibits the use of HHGE for reproductive purposes’. However, this is unclear. At 463, Spaander notes that ‘terms such as “designer babies”, “trait selection” and “eugenics”… are the foundation of existing legal bans … in various legal frameworks’ (footnotes omitted), citing Council of Europe, *Explanatory Report to the Convention for the protection of Human Rights and Dignity of the Human Being with regard to the Application of Biology and Medicine: Convention on Human Rights and Biomedicine*, Oviedo, 4.IV.199, which I discuss below. I suggest that it is therefore clearer that HHGE for uses such as ‘designer babies’ or ‘trait selection’ may be prohibited.

18 Anthony Wrigley, Stephen Wilkinson and John B Appleby, ‘Mitochondrial Replacement: Ethics and Identity’ (2015) 29 Bioethics 631, 638, using this term in relation to PNT, a form of MRT, discussed below.

19 See further Rosamund Scott and Stephen Wilkinson, ‘Germline Genetic Modification and Identity: the Mitochondrial and Nuclear Genomes’ (2017) 37 Oxford J Legal Stud 886, 896. https://doi.org/10.1093/ojls/gqx012.

20 Council of Europe (n 17) [89] (emphasis added).

21 See eg discussion of eugenics in Jonathan Glover, ‘Eugenics: Some Lessons from the Nazi Experience’ in John Harris and Søren Holm (eds), *The Future of Human Reproduction: Ethics, Choice and Regulation* (OUP 1998) 55; and in Dan Wikler, ‘Can We Learn from Eugenics?’ (1999) 25 J Med Ethics 183.

22 Council of Europe, Committee on Bioethics, ‘Statement on Genome Editing Technologies’ (2015) DH-BIO/INF (2015) 13, 2.

23 EU Charter of Fundamental Rights, Art 3(2)(b) (emphasis added).

24 Explanatory note 3, Official Journal of the European Union C 303/17—14.12.2007.

25 NCOB (n 6) [4.46].

26 WHO (n 5) x.

27 ibid [9].

28 Glenn I Cohen, Jacob S Sherkow and Eli Y Adashi, ‘Handle with Care: *The WHO Report on Human Genome Editing*’ (2022) 52 Hastings Center Report 10, 13.

29 International Commission (n 6) Rec 9.

30 HFE Act 1990 (as amended) ss 3ZA (2)(b), (3)(b) and (4)(b).

31 ibid s 3ZA (4)(c).

32 The Human Fertilisation and Embryology (Mitochondrial Donation) Regulations 2015 (the Regulations); entry into force 29 October 2015.

33 HFE Act 1990 (as amended) s 2.

34 See eg repeated references to the term ‘treatment’ in DH (n 13).

35 HFE Act 1990 (as amended) Sched 2, [1(1)] and [1(1)(b)] respectively. The relevant criteria for PGT are in Sched 2, [1ZA] and [1ZB] (emphasis added).

36 ibid s 11(1)(a), (aa), (b) and (c).

37 European Group on Ethics in Science and New Technologies (EGESNT), *Ethics of Genome Editing* (European Commission 2021) 5.

38 Parliamentary Office of Science and Technology (POST) Postnote, *Genome Editing*, Number 541 (November 2016) 2, 4, 12.

39 House of Commons Science and Technology Committee (HCSTC), *Genomics and Genome Editing in the NHS, Third Report of Session 2017–19*, HC 349, 20 April 2018, [100] (emphasis added).

40 Senior Group Leader, The Francis Crick Institute.

41 HCSTC (n 39) Professor Robin Lovell-Badge, Oral evidence, reply to Question 226 (emphases added).

42 ibid, reply to Question 230 (emphases added).

43 ibid, reply to Question 232 (emphases added).

44 Head of Public Policy, Christian Medical Fellowship.

45 HCSTC (n 39) Philippa Taylor, Oral evidence, reply to Question 230.

46 ibid, reply to Question 232 (emphases added).

47 (Then) Programme Leader, Medical Research Council’s Harwell Institute.

48 HCSTC (n 39) Dr Andy Greenfield, Oral evidence, reply to Question 232 (emphasis added).

49 Partner, Hempsons.

50 HCSTC (n 39) James Lawford Davies, Oral evidence, reply to Question 236 (emphasis added).

51 ibid (emphasis added).

52 ibid, reply to Question 247 (emphasis added).

53 ibid, reply to Question 246 (emphases added).

54 ibid, reply to Question 250 (emphasis added).

55 NASEM (n 6) Box S-2 (emphases added), referring to Recommendation 5.1.

56 ibid 17 Box 1-1 (emphasis added).

57 ibid 188 (emphases added). See also 120 regarding parents.

58 International Commission (n 6) respectively 62, 19–20 and eg 65, 72, 104, 125 (emphases added).

59 ibid 98 (emphasis added). The footnote in question is n 1.

60 NCOB (n 6) 142, citing NCOB, *Genome Editing: An Ethical Review* (NCOB 2016) [4.40] (emphases in original, ie NCOB 2016).

61 Eg ‘Category F’ per the International Commission (n 6) 108.

62 NCOB (n 6) [3.40] (emphasis added).

63 ibid [12] (emphasis added).

64 She is one of the authors of Robert Andorno and others, ‘Geneva Statement on Heritable Human Genome Editing: The Need for Course Correction’ (2020) 38 Trends Biotechnol 351, considered below.

65 WHO (n 5) 6, Box 3 (emphasis added).

66 ibid.

67 ibid.

68 EGESNT (n 37) 29.

69 Christopher Gyngell and Julian Savulescu, ‘The Simple Case for Germline Gene Editing’ (2017) Genes for Life 28, 32.

70 ibid 37.

71 ibid 34.

72 For a review of claims in earlier ethics literature, and the media, see Rulli (n 4) 1074–76.

73 Andorno and others (n 64) 351.

74 ibid 352 (emphases added).

75 ibid (emphases added).

76 ibid (emphases added).

77 Marcy Darnovsky and Katie Hasson, ‘CRISPR’s Twisted Tales: Clarifying Misconceptions about Heritable Genome Editing’ (2020) 63 Perspect Biol Med 155, 158 (emphases added).

78 Rulli (n 4) 1073 (emphases added).

79 Thomas Douglas and Katrien Devolder, ‘Gene Editing, Identity and Benefit’ (2022) 72 Philos Quart 305.

80 This is the view that ‘each person has this distinctive necessary property: that of having grown from the particular pair of cells from which this person in fact grew’. Derek Parfit, *Reasons and Persons* (OUP 1984) 352.

81 Douglas and Devolder (n 79) 310.

82 As Derek Parfit has observed, it is plausible to regard existence itself as a ‘good’ for the person (provided a life is worth living): Parfit (n 80) 358. See also Appendix G in which he notes, 489: ‘Causing someone to exist is a special case because the alternative would not have been worse for this person …. [F]or this reason, causing someone to exist cannot be *better* for this person. But it may be *good* for this person’ (emphasis in original; footnote citing Jeff McMahon omitted). This does not mean that non-existence ‘would have been bad for’ this person. ibid.

83 See discussion using these principles in ibid Chapter 16.

84 Christopher Gyngell, Tom Douglas and Julian Savulescu, ‘The Ethics of Germline Gene Editing’ (2017) 34 J Appl Philos 498, 499 (emphasis added).

85 ibid 499–500 (emphases added).

86 ibid 500. But see also Julian Savulescu and Marcos Alonso, ‘Is Gene Editing Harmless? Two Arguments for Gene Editing’ (2022) Am J Bioethics 23 arguing largely against Sparrow (n 88).

87 Douglas and Devolder (n 79) 310.

88 Robert Sparrow, ‘Human Germline Genome Editing: On the Nature of Our Reasons to Genome Edit’ (2021) Am J Bioethics 1, 7 (emphasis in original).

89 ibid (emphasis in original).

90 ibid 6 (emphasis in original).

91 ibid (emphasis in original). The exception would be where the editing is so extensive that it changes the numerical, rather than the qualitative, identity of the individual. For discussion of numerical versus qualitative identity in the context of MRTs and HHGE, see Scott and Wilkinson (n 19) 904–11.

92 César Palacios-González, ‘Reproductive Genome Editing Interventions are Therapeutic, Sometimes’ (2021) 35 Bioethics 557, 559 (italic presentation of original removed).

93 ibid 559.

94 Palacios-González (n 92) 560, eg if a couple created a set of embryos which were then frozen and the woman is later informed that ‘she is homozygous for a dominant monogenetic disease of late-onset’, ibid.

95 Douglas and Devolder (n 79) 316.

96 See eg discussion of the disability critique in relation to MRTs in Wrigley, Wilkinson and Appleby (n 18); and, in relation to HHGE, in Giulia Cavaliere, ‘Genome Editing and Assisted Reproduction: Curing Embryos, Society or Prospective Parents?’ (2018) 21 Med Health Care Philos 215.

97 This is implicit in the UK regulation of IVF under the HFE Act (as amended) which permits embryos to be discarded; the ECtHR has not held that the embryo has a right to life under Art 2: see eg *Vo v France*, App no 53924/00, Judgment, 8 July 2004, discussed further below.

98 See eg Allen Buchanan and others, *From Chance to Choice: Genetics and Justice* (Cambridge University Press 2000) 214ff.

99 Tom Shakespeare, ‘“Losing the Plot?” Medical and Activist Discourses of Contemporary Genetics and Disability’ (1999) 21 Soc Health Illness 669, 681. See also Rosamund Scott, *Choosing between Possible Lives* (Hart Publishing 2007) for relevant ethical and legal argument.

100 Gyngell and Savulescu (n 69) 33.

101 Cavaliere (n 96) 219.

102 For a more detailed description, see DH (n 13) 5.

103 Wrigley, Wilkinson and Appleby (n 18) 635 (emphasis added).

104 ibid 638.

105 ibid 637.

106 ibid 638.

107 César Palacios-González, ‘Are There Moral Differences Between Maternal Spindle Transfer and Pronuclear Transfer?’ (2017) 20 Med Health Care Philos 503.

108 For discussion of the relevant senses of identity see Scott and Wilkinson (n 19) 904–5.

109 For discussion of this concern, see eg Robert Ranisch, ‘Germline Genome Editing Versus Preimplantation Genetic Diagnosis: Is There a Case in Favour of Germline Interventions?’ (2020) 34 Bioethics 60, 65.

110 See eg Buchanan and others (n 98) 202.

111 Robert Sparrow, ‘Better than Men?: Sex and the Therapy/Enhancement Distinction’ (2010) 20 Kennedy Institute Ethics J 115.

112 In text supported by n 42 (emphases added).

113 NASEM (n 6) 9.

114 ibid 13, Box S-2 (emphases added).

115 International Commission (n 6) 7.

116 Written evidence submitted by the Progress Educational Trust (GNH0014) (October, 2017) [39] (emphases added). Note also the EGESNT’s finding to this effect (n 37) 29.

117 For an account see International Commission (n 6) 22.

118 ibid 1, for first use of the concept in this report.

119 ibid Box S-1, Rec 4(2).

120 See eg ibid, 92, 95, 131; WHO (n 5) Box 3, p 6; NCOB (n 6) [2.12]; NASEM (n 6) 119.

121 HFE Act 1990 (as amended) s 3ZA (2)(b), (3)(b) and (4)(b).

122 Andorno and others (n 64) 352.

123 Darnovsky and Hasson (n 77) 158.

124 Andorno and others (n 64) 352 (emphases added).

125 President’s Council on Bioethics, *Reproduction and Responsibility: The Regulation of New Biotechnologies* (PCBE 2004) 109 (first emphasis in original; second added).

126 International Commission (n 6) 98.

127 Note eg that the EGESNT (n 37) considers that the ‘distinctions between therapy, prevention and enhancement … can be helpful for weighing potential benefits and harms’, 30.

128 Rulli (n 4) 1077.

129 ibid 1076.

130 ibid 1077.

131 ibid 1073.

132 Andorno and others (n 64) 352.

133 Rulli (n 4) 1078.

134 NASEM (n 6) 7; and Rec 5-1, 134.

135 ibid 8, n 5.

136 Rulli (n 4) 1078.

137 Andorno and others (n 64) 353.

138 NASEM (n 6) 113.

139 ibid.

140 ibid 114.

141 ibid 115.

142 ibid 188 (emphases added).

143 NCOB (n 6) [1.43].

144 ibid [2.35].

145 ibid [3.12].

146 ibid [3.45] (emphasis added).

147 International Commission (n 6) 99 (emphasis added).

148 ibid 10.

149 See ‘Child Adoption, Adoption Assessment’ at https://www.gov.uk/child-adoption/adoptionassessment (last accessed 25 February 2024).

150 S 46(6) Adoption and Children Act 2002.

151 Department for Education (DE), *Adoption Strategy: Achieving Excellence Everywhere* (July 2021) [94].

152 Lucy Blake, Martin Richards and Susan Golombok, ‘The Families of Assisted Reproduction and Adoption’ in Françoise Baylis and Carolyn McLeod (eds), *Family-making: Contemporary Ethical Challenges* (OUP 2014) 64–85, 77.

153 HFE Act 1990 (as amended) s 13(6C).

154 See the ‘Register of Information’ in the HFE Act 1990 (as amended) s 31, in particular s 31ZA ‘Request for Information as to Genetic Parentage’, and especially s 31ZA(1), (2)(a), (4) and (5). Regarding possible change, see the Human Fertilisation and Embryology Authority (HFEA) consultation *Modernizing the Regulation of Fertility Treatment and Research involving Human Embryos* at https://www.hfea.gov.uk/media/y3xdctuq/modernising-the-act-consultation.pdf (last accessed 25 February 2024), and its *Modernising Fertility Law: Recommendations from the Human Fertilisation and Embryology Authority (HFEA) for changes to the Human Fertilisation and Embryology Act 1990 (as amended)* (HFEA 2023) at https://www.hfea.gov.uk/about-us/modernising-the-regulation-of-fertility-treatment-and-research-involving-human-embryos/modernising-fertility-law/ (last accessed 25 February 2024). Proposal 8 states: ‘The Act should be amended to enable the removal of donor anonymity from the birth of any child born from donation.’

155 On prospective parents’ potential concerns more generally, see NCOB, *Donor Conception: Ethical Aspects of Information Sharing* (NCOB 2013) eg [4.8], [4.9], [4.33]–[4.40].

156 See Rosamund Scott, ‘New Reproductive Technologies and Genetic Relatedness’ (2024) 87 Mod Law Rev 280. https://doi.org/10.1111/1468-2230.12844.

157 NCOB (n 6) Rec 3.

158
*ACB v Thomson Medical Pte Ltd and others* [2017] SGCA 20.

159 ibid respectively [128] and [129].

160
*Evans v United Kingdom (Evans)*, App no 6339/05, Judgment, 10 April 2007 [72]. Art 8 states, in relevant part: ‘(1) Everyone has the right to respect for his private and family life … (2) There shall be no interference … with … this right except such as is in accordance with the law and is necessary in a democratic society … for the protection of health or morals, or for the protection of the rights and freedoms of others.’

161
*Costa and Pavan v Italy*, App no 54270/10, Judgment, 28 August 2012.

162 See also Spaander (n 17) 477.

163
*Handyside* v *United Kingdom*, App no 5493/72, Judgment, 7 December 1976 [48]–[50].

164
*SH and Others v Austria (SH)*, App no 57813/00, Judgment, 3 November 2011. See further Rosamund Scott, ‘Reproductive Health: Morals, Margins and Rights’ (2018) 81 Mod Law Rev 422, 437. https://doi.org/10.1111/1468-2230.12340.

165
*SH* (n 164) [82].

166 ibid [94] citing *Evans* (n 160) [77].

167 ibid [97].

168 ibid [118].

169 ibid [96]. See further Scott (n 164) 437–39.

170 ibid [113].

171 Spaander (n 17) 465.

172 See text supported by n 10 and n 11 above.

173
*SH* (n 164) [101]. See further Scott (n 164) 445.

174
*Vo v France* (n 97).

175 See discussion of *A, B & C v Ireland*, App no 25579/05, Judgment, 16 December 2010, in Scott (n 164) 429–36.

176 ‘The potentiality of that being and its capacity to become a person … require protection in the name of human dignity, without making it a “person” with the “right to life” for the purposes of Article 2.’ *Vo v France* (n 97) [84].

177
*Parrillo v Italy*, App no 46470/11, Judgment, 27 August 2015 [174].

178 George Letsas, ‘Two Concepts of the Margin of Appreciation’ (2006) 26 Oxford J Legal Stud 705, 729; Eyal Benvenisti, ‘Margin of Appreciation, Consensus and Universal Standards’ (1998–99) 31 New York Univ J Int Law Polit 843, 847.

179 See text supported by n 11 above.

180 WHO Expert Advisory Committee on Developing Global Standards for Governance and Oversight of Human Genome Editing, *Human Genome Editing: A Draft Framework for Governance* (World Health Organization 2020) [28].

181 The term cannot be found in WHO (n 5). Compare Spaander (n 17) 483 regarding the importance of ‘a coherent legal framework, based on human rights, particularly dignity’.

182 Peter FR Mills, ‘Genome Editing and Human Reproduction: The Therapeutic Fallacy and the “Most Unusual Case”’ (2020) 63 Perspect Biol Med 126, 136.

183 See eg David Wiggins, ‘Claims of Need’ in *Needs, Values, Truth* (3rd edn, OUP 1998) 1, 7–9.

184 International Commission (n 6).

185 ibid 101.

186 ibid 102.

187 ibid 114–16.

188 ibid 112.

189 ibid 116.

190 Andorno and others (n 64) 352.

191 Rulli (n 4) 1079.

192 Darnovsky and Hasson (n 77) 160.

193 International Commission (n 6) 112.

194 ibid 9–10.

195 ibid 108.

196 Douglas and Devolder (n 79) 316.

197 Respectively, HFE Act (as amended) s 13(9)(c) and s 13(10)(c) and HFEA, *Code of Practice* (Edition 9.4, revised October 2023), ‘Interpretation of mandatory requirements 10C’.

198 ibid [10.19].

199 s 13(5) of the HFE Act 1990 (as amended) states: ‘A woman shall not be provided with treatment services unless account has been taken of the welfare of any child who may be born as a result of the treatment (including the need of that child for supportive parenting), and of any other child who may be affected by the birth.’

200 Emily Jackson, ‘Conception and the Irrelevance of the Welfare Principle’ (2002) 65 Mod Law Rev 176.

201 See Derek Parfit’s work on the ‘non-identity problem’ (n 80) 35. See further n 230 below.

202 Sparrow (n 88) 6; Ranisch (n 109) 65; Rulli (n 4) 1078: HHGE is a ‘selective … technology’.

203 International Commission (n 6) 4, Rec 6. This also recommends ‘long-term follow-up of resulting children and adults’.

204 Parfit (n 80) 358. See further text supported by n 201 above.

205 International Commission (n 6) 97–99. The term is found in: Institute of Medicine, National Academies of Sciences, Engineering, and Medicine, *Mitochondrial Replacement Techniques: Ethical, Social, and Policy Considerations* (National Academies Press 2016) 116.

206 NCOB (n 6) [12], Principle 1 (emphases added).

207 HFEA (n 197) [7.1]–[7.6].

208 Andorno and others (n 64) 352.

209 International Commission (n 6) 102.

210 ibid 103.

211 ibid.

212 ibid 117.

213 ibid 10.

214 ibid 48.

215 ibid 50.

216 ibid 48.

217 ibid 51.

218 ibid 50.

219 See discussion in eg HGC, *Making Babies: Reproductive Decisions and Genetic Technologies* (2006) [4.10].

220 International Commission (n 6) 104.

221 ibid 118. For a supportive approach to prospective parents’ interests and useful discussion of various ethical issues, see Guido de Wert and others, ‘Responsible Innovation in Human Germline Gene Editing: Background Document to the Recommendations of ESHG and ESHRE’ (2018) 26 Eur J Human Genet 450.

222 International Commission (n 6) 109.

223 ‘Polar bodies are cells produced as an oocyte progresses through the meiotic divisions’. International Commission (n 6) 51. Polar-body genotyping ‘has the potential to identify zygotes that have inherited from the mother an allele that causes a dominant monogenic disease’. ibid 109. See further 51–53.

224 ibid 109. See further 51–53.

225 ibid 109.

226 On ‘disability ethics’ see eg Rulli (n 4) 1081; for discussion of the disability critique as regards HHGE versus PGT, see Cavaliere (n 96).

227 See eg WHO, ‘Infertility’ at https://www.who.int/health-topics/infertility#tab=tab_2 (last accessed 25 February 2024); and David Orentlicher, ‘Societal Disregard for the Needs of the Infertile’ in Leslie Francis (ed), *The Oxford Handbook of Reproductive Ethics* (OUP 2017) 357–87.

228 See eg NCOB (n 6) Principle 2 regarding ‘social justice and solidarity’; but note that HHGE for serious genetic conditions seems unlikely to be the main concern here. I have discussed various other issues in Rosamund Scott, ‘Governance Challenges for Heritable Human Genome Editing: Contested Concepts and Lines’ in Derek Beyleveld, Roger Brownsword and Marcus Duewell (eds), *Research Handbook on Law, Governance and Bioethics* (Edward Elgar 2024 forthcoming).

229 That is, on a person-affecting perspective.

230 As noted earlier (in text supported by nn 198–201), as regards HHGE a concern with the health and well-being of a future child may be thought to be consistent with the current regulatory concern as to the welfare of the future child expressed in s 13(5) of the amended Act. However, given legitimate critique of that section as regards IVF per se noted earlier, and given that if HHGE were legalised it would be accompanied by detailed regulations supported by new sections of the HFEA *Code of Practice*, it remains doubtful that s 13(5) should be retained. Any difficult cases relating to IVF per se could be addressed, for instance, by a clinical ethics committee, as recommended by the House of Commons Science and Technology Committee in its report *Human Reproductive Technologies and the Law* (Fifth Report of Session 2004-5, Volume 1) [107]. As regards the welfare of a future gene-edited child, some regulatory oversight is appropriate, notwithstanding that it is difficult for a child to complain of being born unless her/his life is of such poor quality that it is not worth living (see generally Parfit (n 80)), and a ‘not worth living’ standard is unlikely to be regarded as acceptable for novel ARTs. As we have seen, a concern with the welfare of the future child is expressed in various reports discussed here, eg NCOB (n 6) and the International Commission (n 6).

231 Regarding PGT, see eg HFEA (n 197) [10.5], [10.6] and [10.9].

